# A Manual of Auscultation and Percussion

**Published:** 1849-01

**Authors:** 


Art. VI.—*A Manual of Auscultation and Percussion.* By M. M. Barth and Roger—translated, with additions, by Francis G. Smith, M. D. Philadelphia, Lindsay & Blakiston, 1845. pp 160—12 mo.

This manual, which, as is set forth in the preface of the American edition, is a translation of the *resumé* of the second edition of Barth & Roger's work on auscultation, with the addition of a new treatise or Percussion by the same authors, cannot fail to be acceptable to the profession and particularly to the younger part of it in the United States.

A hand-book of this kind should be in the hands of every student, more especially as so little time is allowed in most of the medical schools of our country for the special study of the science of which it treats.

Not only is auscultation of daily and inestimable service in the diagnosis of diseases of the respiratory and circulatory apparatus, but it lends valuable aid to surgery, in the diagnosis of fractures; to midwifery, in the diagnosis of pregnancy; is applied to certain diseases of the brain, and not very long since was reported to have been practiced with the happiest results in inflammation of the peritoneum. The medical press has furnished us with the results of the labors in this department not only of many of our own physicians, in the shape of treatises and brief *Vade mecums*, but has added also reports and translations, until we need be at no loss for either monographs or compilations, home or foreign, on this subject. But among all these, we are acquainted with no work better calculated to fulfil the end which it proposes than the one before us. We are in the habit of recommending it to our students, and now extend the recommendation to the readers of the Journal. D. W. Y.

